# Altered gut microbiota and metabolite profiles in community-acquired pneumonia: a metagenomic and metabolomic study

**DOI:** 10.1128/spectrum.02639-24

**Published:** 2025-03-10

**Authors:** Fuxin Zhang, Jiahui Luan, Lijun Suo, Haiyan Wang, Yi Zhao, Tianyu Sun, Yawen Ni, Hongyun Cao, Xiaohui Zou, Bo Liu

**Affiliations:** 1 Department of Clinical Microbiology, Zibo City Key Laboratory of Respiratory Infection and Clinical Microbiology, Zibo City Engineering Technology Research Center of Etiology Molecular Diagnosis, Zibo Municipal Hospital, Zibo, China; 2 Department of Pulmonary and Critical Care Medicine, Zibo Municipal Hospital, Zibo, China; 3 National Center for Respiratory Medicine, Chinese Academy of Medical Sciences, Beijing, China; 4 State Key Laboratory of Respiratory Health and Multimorbidity, Chinese Academy of Medical Sciences, Beijing, China; 5 National Clinical Research Center for Respiratory Diseases, Chinese Academy of Medical Sciences, Beijing, China; 6 Institute of Respiratory Medicine, Chinese Academy of Medical Sciences, Beijing, China; 7 Center of Respiratory Medicine, China-Japan Friendship Hospital, Beijing, China; 8 Peking Union Medical College, Chinese Academy of Medical Sciences, Beijing, China; 9 Changping Laboratory, Beijing, China; 10 Department of Weifang People’s Hospital, Shandong Second Medical University 372527 , Weifang, China; 11 Department of Pulmonary and Critical Care Medicine, Shandong Institute of Respiratory Diseases, The First Affiliated Hospital of Shandong First Medical University, Shandong Provincial Qianfoshan Hospital, Shandong University, Jinan, China; University of Kentucky, Lexington, Kentucky, USA

**Keywords:** community-acquired pneumonia, gut microbiota, metabolomics, bile acids, short-chain fatty acids

## Abstract

Emerging evidence suggests that altered gut microbiota is linked to community-acquired pneumonia (CAP), but the potential mechanisms by which gut microbiota and its metabolites contribute to the development of CAP remain unclear. Fecal samples from 32 CAP patients and 36 healthy controls were analyzed through metagenomic sequencing and metabolomic profiling. The gut microbiota composition in CAP patients showed significant differences and lower diversity compared to healthy controls. Genera involved in short-chain fatty acid (SCFA) production, such as *Faecalibacterium*, *Ruminococcus*, and *Eubacterium*, as well as species like *Faecalibacterium prausnitzii*, *Bifidobacterium adolescentis*, *Eubacterium rectale*, *Prevotella copri*, and *Ruminococcus bromii*, were significantly depleted in CAP patients. Bacterial co-occurrence network analysis revealed an over-representation of pro-inflammatory bacteria, which contributed to the core gut microbiome in CAP patients. Metabolomic analysis of fecal samples identified a distinct metabolic profile, with a notable increase in arachidonic acid, but a decrease in secondary bile acids, such as deoxycholic acid, lithocholic acid, and ursodeoxycholic acid, compared to healthy controls. Spearman correlation analysis between differential microbiota and bile acids showed that *Faecalibacterium prausnitzii*, *Bifidobacterium adolescentis*, *Eubacterium rectale*, and *Prevotella copri* were positively correlated with ursocholic acid, lithocholic acid, and ursodeoxycholic acid, respectively. Our results suggest that the reduction in secondary bile acids, insufficient production of SCFAs, and an overabundance of pro-inflammatory bacteria may contribute to metabolic inflammation in the body. These factors could play a key role in the pathogenesis of CAP, driven by gut microbiota alterations.

**IMPORTANCE:**

This study presents a comprehensive metagenomic and metabolomic analysis of fecal samples from community-acquired pneumonia (CAP) patients, identifying key characteristics, such as decreased secondary bile acids, imbalanced short-chain fatty acid production, and increased pro-inflammatory bacteria. These findings provide valuable insights into the mechanisms linking gut microbiota alterations to CAP pathogenesis and suggest that targeting the gut microbiota could be a promising strategy for intervening in CAP.

## INTRODUCTION

Community-acquired pneumonia (CAP) is one of the most common infectious diseases causing high morbidity and mortality globally, especially among children and the elderly (1, 2). According to the Global Burden of Disease Study, 489 million people suffered from lower respiratory infections in 2019 (3). CAP continues to be a major concern related to public health around the world in the post-COVID-19 era.

Accumulating evidence highlights lung diseases, especially infections, can influence the composition and function of the gut microbiota, which plays a crucial role in lung immunity and host defense against respiratory infections (4, 5), referred to as the gut-lung axis. Dysfunction of gut microbiota negatively impacts the regulation of the gut-lung axis and is associated with an increased susceptibility to respiratory infections (6). For example, early-life antibiotic exposure has pronounced effects on gut microbiome development and selection of antimicrobial resistance, which can increase susceptibility to pulmonary viral infections (7, 8). Bacteroides-dominant microbiota profiles were more susceptible to bronchiolitis (9). However, the relationship between CAP and gut microbiome was unclear.

Emerging studies have indicated that commensal bacteria and their microbiota-derived metabolites, such as short-chain fatty acids (SCFAs) and secondary bile acids (SBAs), could regulate host immunity (10). These substances can modulate host inflammation by regulating host inflammation and T cell differentiation and support immune homeostasis by promoting defense against multiple pathogens (11–14).

Furthermore, the reduced abundance of butyrate-producing commensal bacteria increased the risk of lower respiratory infections in patients undergoing allogeneic hematopoietic stem cell transplantation (15). Studies in mice demonstrated that microbial metabolite desaminotyrosine can enhance type I IFN signaling against influenza (16). These findings suggested that the imbalance of the intestinal microbiome and associated metabolites may be related to immune responses to respiratory infections.

In recent years, several observational studies focusing solely on the gut microbiome have demonstrated significant changes in the microbiome characteristics of CAP patients. Given that microbiota-derived metabolites act as intermediaries between the intestinal microbiome and host immunity, comprehensive analyses of the intestinal microbiome and metabolome could offer valuable insights into the mechanisms underlying the development of CAP.

This study presents a comprehensive metagenomic and metabolomic analysis of fecal samples from CAP patients and healthy controls. The results showed that dysbiosis of the gut microbiota can lead to decreased SBAs, insufficient production of SCFAs, and an overabundance of pro-inflammatory bacteria, ultimately leading to metabolic inflammation.

## MATERIALS AND METHODS

### Participants

All eligible participants or their legal representatives provided written informed consent. From 20 January to 29 February 2023, we recruited 32 individuals with CAP, who were hospitalized patients aged ≥18 years. Since elderly people have a high incidence of pneumonia, the enrolled patients were older, about 65 years old. The diagnosis of CAP was based on the diagnosis and treatment guidelines for CAP by the Infectious Diseases Society of America and the American Thoracic Society (17). Clinical data, including age, sex, white blood cell, smoking, pathogen information, pneumonia severity index, and mechanical ventilation, were obtained from hospital electronic medical records. In addition, 36 healthy volunteers of comparable age without acute infection were recruited as controls.

### Sample collection

Fecal samples were collected from CAP patients within 24 hours of admission before treatment (named CAP groups) and healthy controls. Fresh fecal samples were aseptically collected from each participant. The outer surface of the feces was discarded, and the internal matter was collected in sterile containers, which were then divided into two equal portions. Then, the samples were immediately stored in a −80°C freezer within 1 hour for microbiome and metabolome analyses.

### Targeted next-generation sequencing for pathogen detection

Sputum samples were collected from all CAP patients (if there is no sputum, oropharyngeal swabs were an alternative) after initial admission for targeted next-generation sequencing (tNGS). Respiratory Pathogen Detection Kit (KingCreate, Guangzhou, China) was used following the manufacturer’s protocol. Briefly, nucleic acid was extracted, and a set of 198 microorganism-specific primers (detailed pathogen lists are given in Data S1) were employed to perform ultra-multiplex PCR amplification. The qualified library was sequenced on an Illumina MiniSeq platform. The tNGS results were ultimately evaluated by three experienced respiratory physicians to confirm the patient’s potential pathogen.

### DNA extraction, metagenomic sequencing, and quality control

Total genomic DNA from stool specimens was extracted using the OMEGA Mag-Bind Soil DNA Kit (M5635-02) (Omega Bio-Tek, Norcross, USA). The purity and concentration of the extracted DNA were then measured by a Qubit 4 Fluorometer and agarose gel electrophoresis, respectively. Metagenomic shotgun sequencing libraries with insert sizes around 400 bp were constructed according to the Illumina TruSeq DNA Sample Preparation Guide (Illumina, USA). The library preparations were sequenced using the Illumina HiSeq 4000 platform with a pair-end length of 150 bp (Illumina, San Diego, CA, USA).

FastQC (version 0.11.8) was used to quality control raw reads, and the quality control results of each sample file were summarized using MultiQC (version 1.8). Trimmomatic was used to remove adapter contamination and filter low-quality reads. Subsequently, kneaddata was used to identify and remove the human genome. After filtering, the remaining high-quality paired sequences were used for taxonomic profiling utilizing MetaPhlan2. Functional annotation of the metagenome was performed using the KEGG Orthology database (18) and the Comprehensive Antibiotic Research Database, with ≥90% sequence identity and ≥90% protein coverage.

### Metagenomics analysis

Gut microbiome alpha-diversity and beta-diversity analyses were conducted using the R package vegan to calculate the diversity of microbial taxa between the two groups. The differences in microbial structure and composition of each group were assessed through principal coordinate analysis (PCoA) based on Bray-Curtis distance. Linear discriminant analysis of effect size was applied to assess statistically significant differential species using the microeco package in R. The 50 most abundant species or genera were selected for differential abundance analysis. Spearman’s correlation analysis was used to reveal the taxonomic relationship at the species level. Gephi was applied to visualize the network. Global transitivity, eigenvector centrality, and weighted closeness were used to evaluate the importance of hub species in the network. Eigenvector centrality refers to the sum of connectivity between a node and its neighboring nodes, which can be used to measure the influence and importance of the node in a network. Global transitivity refers to the global clustering coefficient of the network graph, which measures the degree of connectivity between nodes and indicates the network’s spreadability. The high clustering coefficient is associated with network degradation and can reduce the stability of the network. Weighted closeness refers to the reciprocal of the sums of the shortest distances between a node and all other nodes. In microbial networks, the greater the density of microorganisms, the greater their influence on their environment. Finally, we used the selbal (19) package to identify a microbial signature for CAP disease that is able to discriminate between CAP and non-CAP individuals. Selbal is a greedy stepwise algorithm for the selection of taxa balances or microbial signatures to predict disease status.

### Metabolomic samples’ extraction and data analysis

Fifty milligram fecal samples were added to 500 µL of extraction buffer (methanol: acetonitrile:water = 2:2:1 [vol/vol]) in labeled tubes in a low-temperature environment. The mixture was treated with high-speed homogenizer (JXFSTPRP-24, Shanghai Jingxin Technology Co., Ltd.) at 35 Hz for 4 min and then processed with a high-throughput tissue crusher (PS-60AL, Shenzhen Leaderbang Electronic Co., Ltd.) for 5 min in an ice bath. The treated samples were left at −40°C for 1 hour and then centrifuged at 12,000 rpm for 15 min using microcentrifuge (Heraeus Fresco17, Thermo Fisher Scientific). A volume of 60 µL of supernatant was added to 2 mL injection bottles, and 10 µL of supernatant from each sample was taken to prepare quality control (QC) samples for machine testing. Metabolites were separated by Waters ACQUITY UPLC BEH Amide column using a high-resolution mass spectrometer (Vanquish, Thermo Fisher Scientific). Mobile phase A is an aqueous solution containing 25 mmol/L of ammonium acetate and 25 mmol/L of ammonium hydroxide. Mobile phase B is acetonitrile.

The Thermo Orbitrap Exploris 120 mass spectrometer was used to collect DDA mass spectrometric data in positive and negative ion modes under the control of Xcalibur software (version: 4.7, Thermo). HESI source, spray voltage 3.5 kV/−3.0 kV; sheath gas, 40 arb; auxiliary gas, 15 arb; capillary temperature, 325°C; auxiliary gas temperature, 300°C; primary resolution, 60,000; scan range, 100–1,000 *m*/*z*; AGC Target Standard, Max IT 100 ms; the top four ions were screened for secondary fragmentation, and dynamic exclusion time was 8 s, secondary resolution was 15,000, HCD collision energy was 30%, and AGC Target Standard was Max IT Auto. All formal samples and QC samples were loaded into the equipment according to the above-mentioned chromatography and mass spectrometry methods. Before the formal injection, QC samples were injected two to four times to balance the system. During the injection process, one QC sample was injected for every 5–10 samples for subsequent data evaluation and quality control.

The metabolomic raw data obtained were converted to mzXML format using MSConvert in the ProteoWizard software package (version 3.0.8789) (20), followed by processing with the XCMS package (21) of R for peak identification, filtration, and alignment. Peaks with relative standard deviation > 30% in QC samples were excluded for the subsequent identification of metabolites. Finally, the metabolites were annotated using the KEGG and HMDB databases to identify the metabolites of interest. Orthogonal partial least square discriminant analysis (OPLS-DA) was used to evaluate metabolomic profile alterations between groups. Metabolites meeting the criteria of having variable projection importance (VIP) > 1, *P*-value < 0.05, and logFC > |1| were ultimately identified as differential expression metabolites. The R package PAPi was used to predict the PAPi score of metabolic pathways based on the abundance of the metabolites (22).

### Statistical analysis

All statistical analyses were performed in the R programming language (version 4.3.2). Differential abundance of metabolites, gut microbial families, species and genera, resistance genes, and KEGG functional pathway levels were assessed using the two-sided Wilcoxon rank-sum test between two groups, and the adjusted FDR *P*-value was calculated using the Benjamini-Hochberg method. Spearman’s correlation analysis was performed to investigate the correlation between the differential gut microbiome and differential metabolites. The ggplot2, pheatmap, ggtree, and microbiomeViz packages were used to draw all box plots, bar plots, heatmaps, and cladograms.

## RESULTS

### Patients’ cohort and clinical characteristics

This study enrolled 32 CAP patients and 36 matched non-infectious healthy controls. The pneumonia severity index of 28 (87.5%) patients was classes I–III, and 4 (12.5%) patients were class IV. Their clinical characteristics are listed in Table 1. For pathogen detection, 24 (75%) patients demonstrated positive results by tNGS. Among them, only virus infection (mainly SARS-CoV 2) accounted for the highest proportion (28.1%), and virus and bacteria (including SARS-CoV 2, *Streptococcus pneumoniae*, *Haemophilus influenzae*, etc.) coinfection occupied 25%. Four patients (12.5%) were diagnosed with bacterial infections only, one patient (3.1%) had fungal infection only, one patient (3.1%) had both viral and fungal co-infection, and one patient (3.1%) had co-infections of virus, bacteria, and fungus. Detailed pathogen information of participants is shown in Table S1.

**TABLE 1:** Clinical characteristics of enrolled patients and controls^*c*^

	CAP patients (*N* = 32)	Controls (*N* = 36)	*P* value
Age (mean ± SD)	65.81 ± 12.9TL ‘. B 6	64.22 ± 5.91	0.509
Sex			
Female, *n* (%)	13 (38.2）	21 (61.8）	0.145
Male, *n* (%)	19 (55.9）	15 (44.1）	
White blood cell (mean ± SD)	6.24 ± 2.38		
Smoke	3/32 (9.3%)		
Complication			
Respiratory diseases^*a*^	6/32 (18.8%)		
Type 2 diabetes mellitus	5/32 (15.6%)		
Hypertension	14/32 (43.6%)		
Coronary artery disease	5/32 (15.6%)		
Pathogen**^*b*^**			
Virus only, *n* (%)	9/32 (28.1%)		
Bacteria only, *n* (%)	4/32 (12.5%)		
Fungus only, *n* (%)	1/32 (3.1%)		
Virus and bacteria, *n* (%)	8/32 (25%)		
Virus and fungus, *n* (%)	1/32 (3.1%)		
Virus, bacteria, and fungus, *n* (%)	1/32 (3.1%)		
Undetected, *n* (%)	8/32 (25%)		
Pneumonia severity index			
Classes I–III, *n* (%)	28/32 (87.5%)		
Class IV, *n* (%)	4/32 (12.5%)		
Mechanical ventilation	2/32 (6.3%)		

^*a*^ Chronic obstructive pulmonary disease and chronic bronchitis.

^*b*^ Detected by targeted next-generation sequencing using sputum or oropharyngeal swab (for those patients who did not have sputum). List of 198 pathogens are shown in Data S1.

^*c*^ *t*-test with two independent samples was used to compare the ages between the two groups, and no statistically significant difference was found (*P* = 0.509). In addition, there was no statistically significant difference in gender composition between the two groups (*P* = 0.145), and the chi-square test was used for analysis.

### Alterations in the gut microbiome of CAP patients

**Fig 1:**
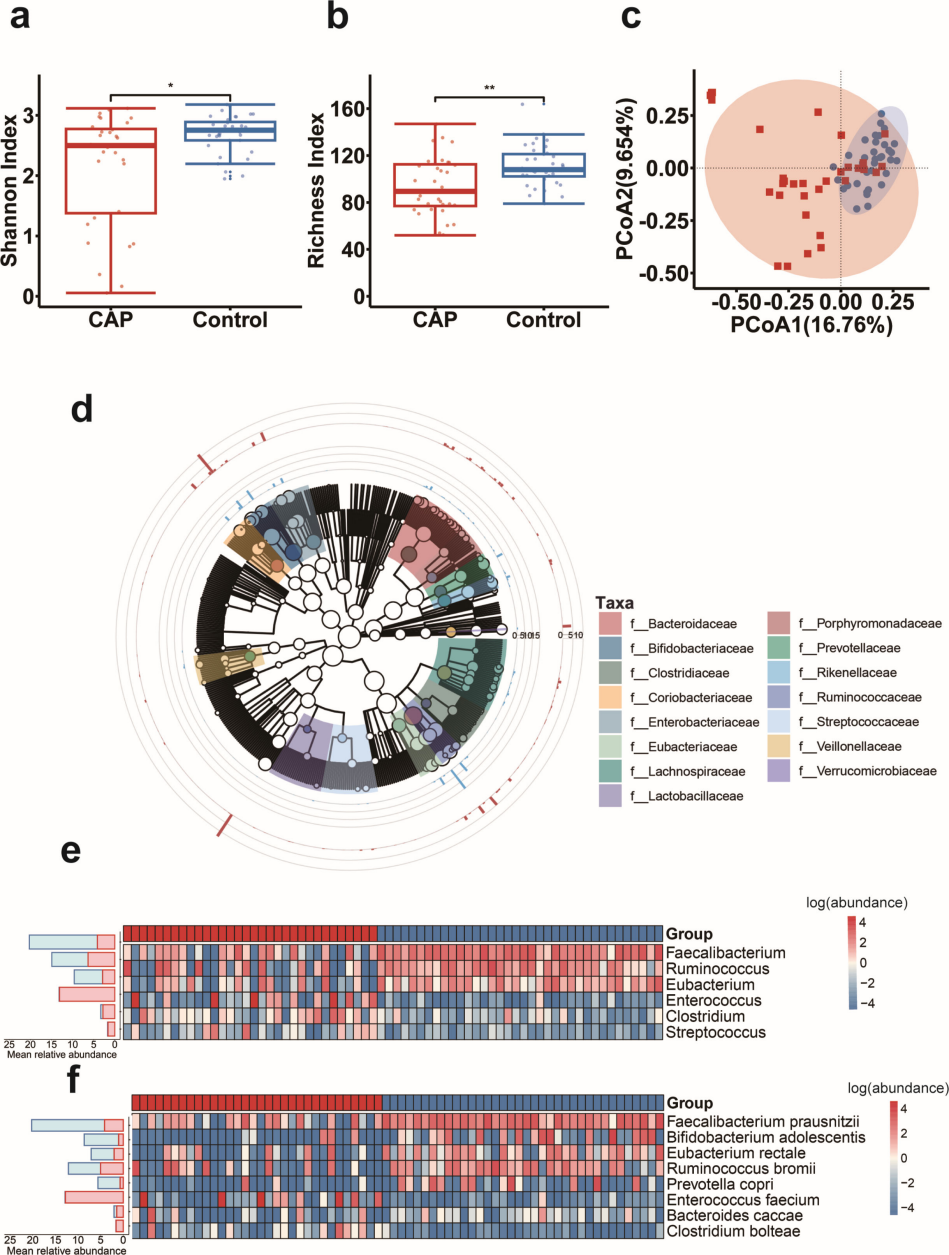
Gut microbiome changes in patients with CAP compared to the control group. (**a**) The box plot showing richness index and (**b**) Shannon index of gut microbiota. (**c**) The PCoA of gut microbial species level. *P* < 0.01. (**d**) Cladogram showing the microbial community profiles in CAP patients (red) and control group (blue). The circles radiating from inside to outside represent the seven taxonomic levels of the kingdom, phylum, class, order, family, genus, and species. The evolutionary tree has been annotated at the family level, with empty circles indicating no annotated species classification and filled circles indicating annotated species classification. (**e**) Heatmap illustrating the differentiated changes in gut microbial genera between CAP patients and the control group. (**f**) Heatmap illustrating the differentiated changes in gut microbial species between CAP patients and the control group. The color gradient displays the abundance changes of different species in the sample; dark red indicates high bacterial abundance, deep blue indicates low abundance, and white indicates intermediate abundance. The stacked bar charts on the side represent the average relative abundance of different gut microbiota genera and species in the CAP patients and controls, respectively.

To explore the characteristics of gut microbiota in patients with CAP, we performed metagenomic sequencing on fecal samples collected from 32 CAP patients and 36 age- and gender-matched non-infectious healthy controls. Gut microbial richness index and Shannon index were lower in patients with CAP compared with control groups (Fig. 1a and b). The beta-diversity of the microbial composition showed a significant difference between the two groups (Fig. 1c). Overall, the abundance of *Enterococcaceae*, *Clostridiaceae*, *Coriobacteriaceae*, and *Streptococcaceae* families in CAP patients increased (Fig. 1d). Furthermore, analysis of the composition of gut microbiota at the genus level revealed that SCFA-producing genera *Faecalibacterium*, *Ruminococcus*, and *Eubacterium* were significantly decreased in the CAP groups. In contrast, the abundance of *Enterococcus*, *Streptococcus*, and *Clostridium* was significantly higher in the CAP groups (FDR *P* < 0.05, Wilcoxon rank-sum test, Fig. 1e).

At the species level, we observed remarkably differentiatial abundance of species corresponding to SCFA production. In the CAP groups, the abundance of *Faecalibacterium prausnitzii*, *Bifidobacterium adolescentis*, *Eubacterium rectale*, *Prevotella copri*, and *Ruminococcus bromii* was significantly reduced, while the abundance of *Enterococcus faecium* and *Clostridium bolteae* was significantly increased (FDR *P* < 0.05, Wilcoxon rank-sum test, Fig. 1f).

### Distinct pattern of bacterial co-occurrence network in CAP patients

**Fig 2:**
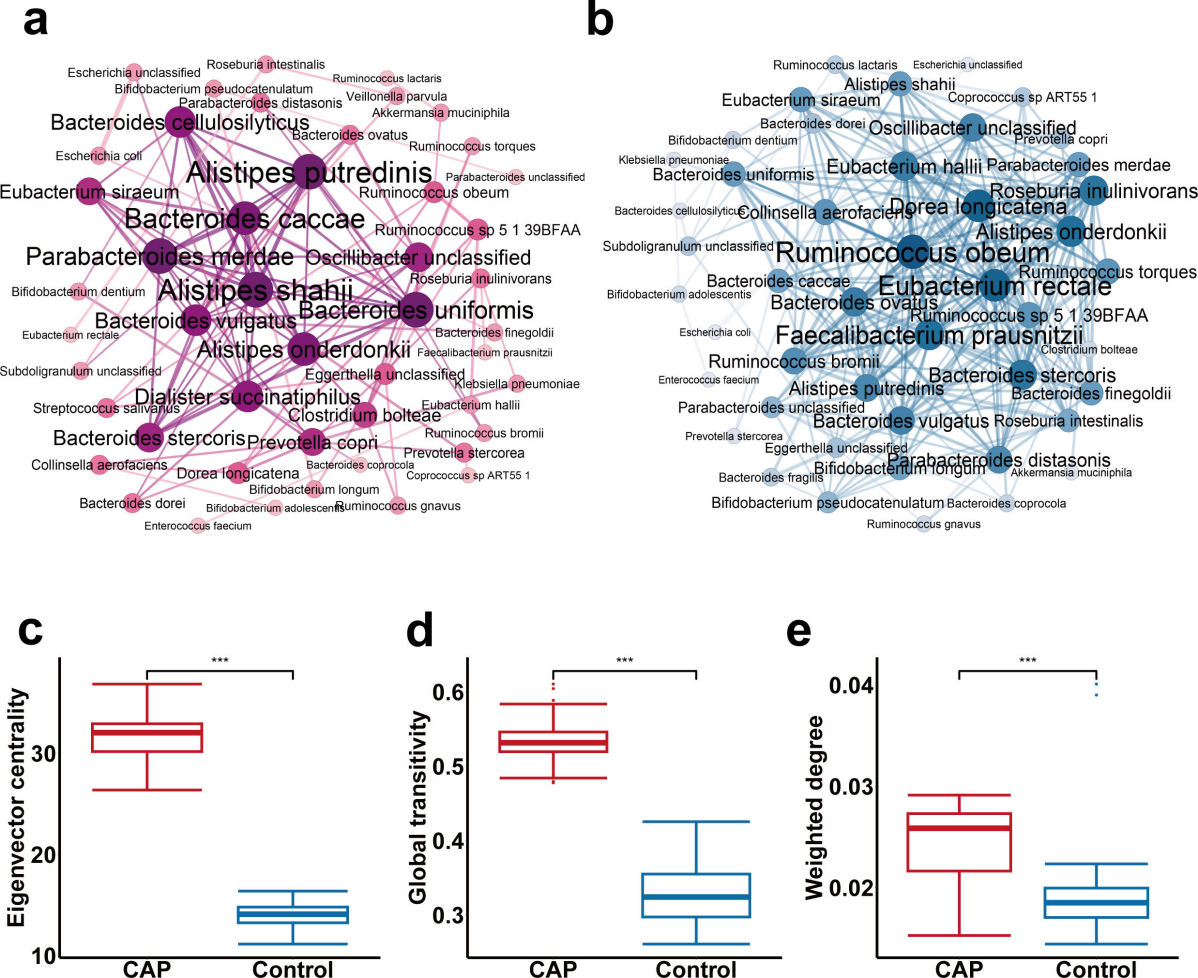
Changes in the co-occurrence network of intestinal bacteria in CAP patients compared to the control group. Co-occurrence network of intestinal bacterial species in CAP patients (**a**) and control group (**b**). Node size and edge width indicate the degree of species and species-species correlation in each network, respectively. Edges with correlation value > 0.3, *P* < 0.05 were retained. (**c**) Eigenvector centrality, (**d**) global transitivity, and (**e**) weighted closeness of individual species in bootstrapped bacterial co-occurrence networks. The bootstrap network was constructed by randomly selecting 30 samples for 100 bootstraps in the CAP and control groups, respectively. ***P < 0.001.

We speculated that the interaction of gut microbiota plays a certain role in the occurrence and development of CAP. Spearman’s correlation analysis on the top 50 most abundant species showed that *Alistipes putredinis* and *Bacteroides caccae* in CAP patients and *Ruminococcus obem*, *Faecalibacterium prausnitzii*, and *Eubacterium rectale* in control groups as the most severe core species (Fig. 2a and b). We further randomly selected 30 individuals from each group for network construction (permutation = 100 times) to verify the symbiotic relationship between bacterial communities. Compared with the control groups, CAP patients showed higher global transitivity (*P* < 0.001), eigenvector centrality score (*P* < 0.001), and weighted closeness (*P* < 0.001) (Fig. 2c through e), indicating that some benign interactions of the gut microbiota were weakened in CAP patients.

In order to further understand the mechanism and function of the core species during the development process of CAP patients in bootstrapped co-occurrence networks, we ranked the weighted degree and weighted proximity of the species in each group of networks, respectively. Significantly, several pro-inflammatory species, such as *Alistipes shahii*, *Bacteroides caccae*, and *Alistipes putredinis*, were more likely to constitute core species of the gut microbiota in CAP patients (Fig. S1a and b), while multiple SCFA-producing species, including *Eubacterium rectale*, *Faecalibacterium prausnitzii*, and *Dorea longicatena* were the core species in the control group (Fig. S1c and d). These data revealed distinct bacterial symbiotic network patterns between CAP patients and control groups. The pro-inflammatory species were the core species in CAP patients’ networks, while the SCFA-producing species were the core bacteria in the control groups’ networks.

### Microbial function changes and expression of antibiotic resistance genes increase in CAP patients

**Fig 3:**
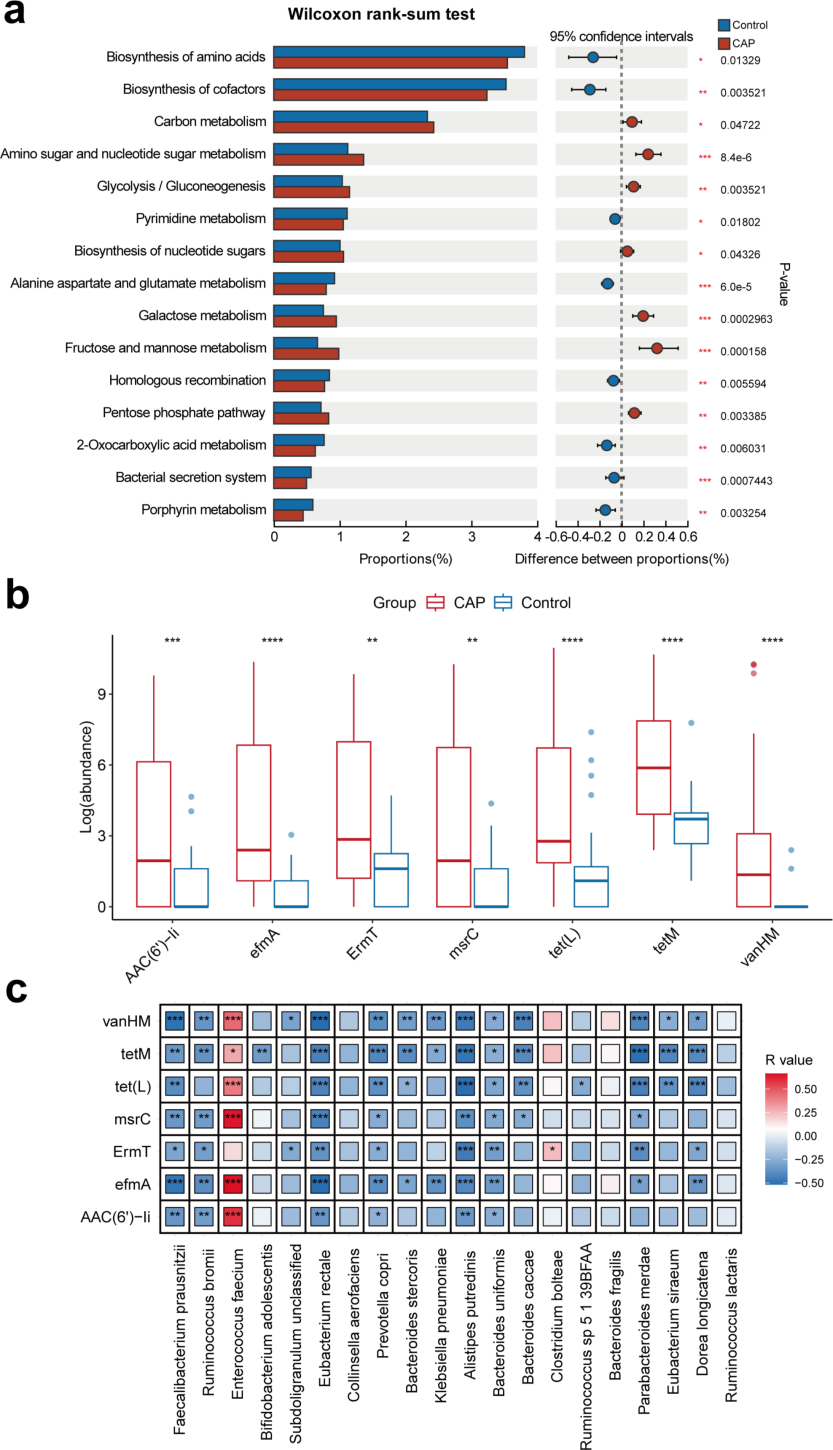
The comparison of microbial function and antibiotic resistance genes (ARGs) between CAP patients and control groups. (**a**) Microbial function characteristics in CAP patients. (**b**) Significant differences in ARGs between the two groups. (**c**) The correlations between gut microbiomes and ARGs at the species level (**b**). *P < 0.05; **P < 0.01; ***P < 0.001; and ****P < 0.0001.

Furthermore, KEGG analysis was conducted to explore the microbial functional characteristics associated with the occurrence of CAP. A total of seven KEGG functional pathways were significantly enriched in CAP patients (FDR *P* < 0.05, Wilcoxon rank-sum test, Fig. 3a), including amino sugar and nucleotide sugar metabolism, carbon metabolism, biosynthesis of nucleotide sugars, glycolysis/gluconeogenesis, fructose and mannose metabolism, galactose metabolism, and the pentose phosphate pathway. In contrast, eight KEGG functional pathways, including biosynthesis of amino acids, biosynthesis of cofactors, pyrimidine metabolism, alanine aspartate and glutamate metabolism, bacterial secretion system, homologous recombination, 2-oxocarboxylic acid metabolism, and porphyrin metabolism, were significantly reduced in CAP patients compared to the control group. It has been reported that the metabolic pathways of biosynthesis of amino acids and alanine, aspartate, and glutamate metabolism can affect the capacity for SCFA production (23). Therefore, our data suggested that a significant decrease or increase in the abundance of specific gut microbiota may lead to decreased production of SCFAs in CAP patients.

Given the influence of antibiotic resistance genes (ARGs) on the treatment of CAP patients, we evaluated the antimicrobial resistance characteristics of the microbial communities in CAP patients and control groups. In our study, seven ARGs, including *AAC(6)-li*, *efmA*, *ErmT*, *msrC*, *tet(L)*, *tetM*, and *vanHM*, were significantly higher in CAP patients (Fig. 3b). Subsequent Spearman correlation coefficient analysis revealed strong correlations between differential species and these seven ARGs (Fig. 3c). Specifically, *Enterococcus faecium* was positively correlated with *efmA*, *msrC*, *tet(L)*, *tetM*, and *vanHM*, while *ErmT* was positively correlated with *Clostridium bolteae*. Several dominant species, such as *Faecalibacterium prausnitzii*, *Eubacterium rectale*, and *Prevotella copri*, showed negative correlations with the seven ARGs. Overall, these results suggest that microbial dysbiosis may be related to the occurrence of ARGs.

### Alteration of metabolomic profiling of feces from CAP patients

**Fig 4:**
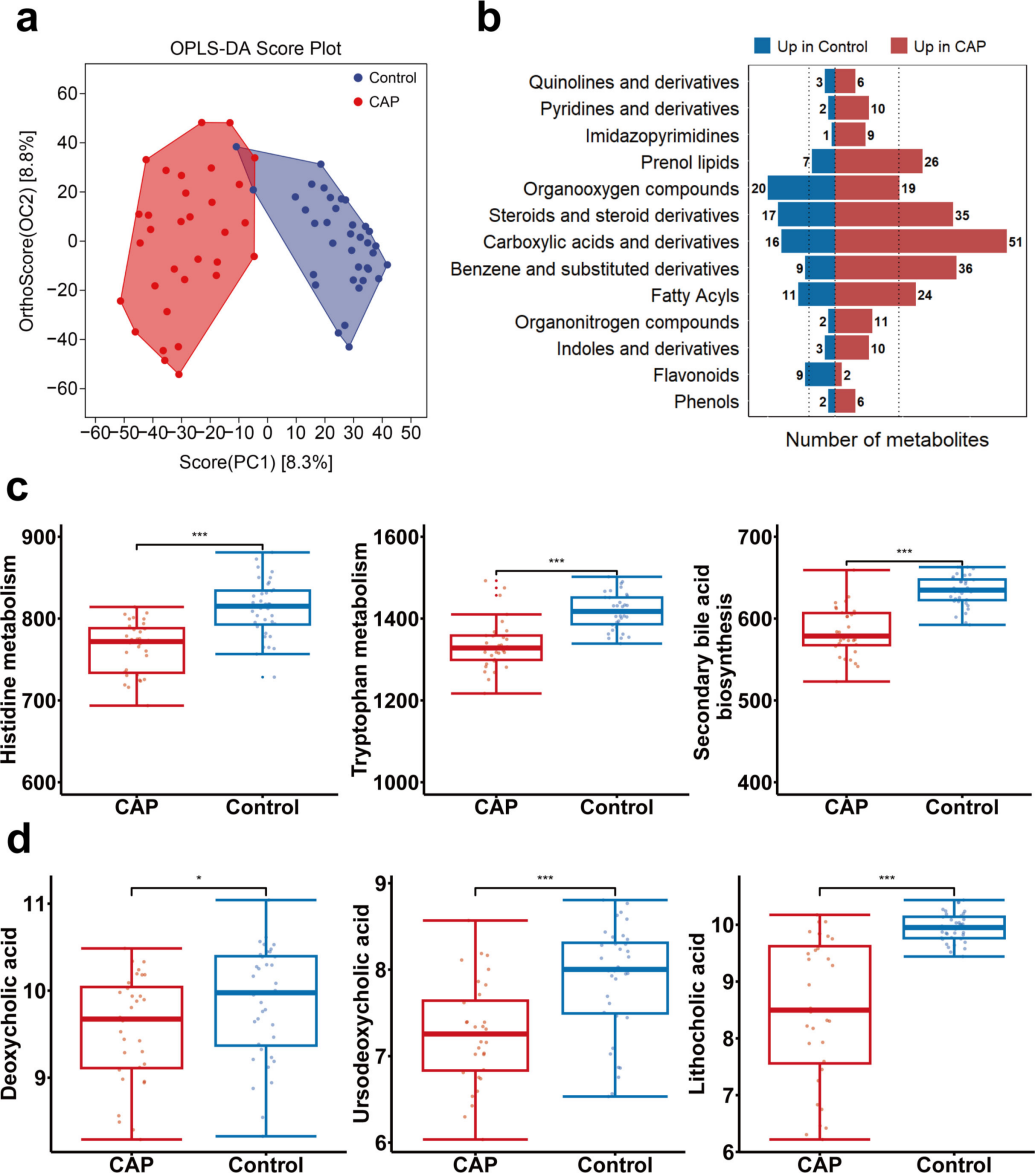
Alterations in fecal metabolites in CAP patients. (**a**) OPLS-DA score plots of fecal metabolites. (**b**) Bar plot of differential metabolites of 13 classes in CAP patients and control group. The dashed lines represent the average number of significantly altered metabolites in the two sets of samples. (**c**) PAPi score of metabolic pathways of histidine metabolism, tryptophan metabolism, secondary bile acid biosynthesis in CAP patients and control group. (**d**) The abundances of deoxycholic acid, lithocholic acid, and ursodeoxycholic acid in CAP patients and control group. *P < 0.05; **P < 0.01; and ***P < 0.001.

Next, to elucidate the possible metabolites involved in the development of CAP, we conducted non-targeted metabolomics on fecal samples collected from 32 CAP patients and 36 non-infectious healthy controls matched for age and gender. OPLS-DA revealed a distinct metabolic profile (Fig. 4a). Among them, 452 metabolites showed significant alterations (*P* < 0.05) in CAP patients (Data S2). The majority of these altered metabolites were classified into the categories of carboxylic acids and derivatives, organooxygen compounds, fatty acyls, and steroids and steroid derivatives (Fig. 4b). VIP values were calculated to assess the importance of each metabolite. We defined the 106 metabolites with VIP > 1 as differential expression metabolites, including 31 upregulated metabolites and 75 downregulated metabolites, as shown in Data S3. PAPi revealed that multiple pathways, including tryptophan and histidine metabolism and secondary bile acid biosynthesis, exhibited substantially lower PAPi scores in patients with CAP compared with the control groups (*P* < 0.05, Fig. 4c).

Based on the results of the fecal microbiome analysis, we further focused on secondary bile acid in the fecal metabolome analysis. The levels of deoxycholic acid, lithocholic acid, and ursodeoxycholic acid were significantly reduced in CAP patients (*P* < 0.05, Fig. 4d). These results indicated a close association between impaired bile acid metabolism and CAP.

### Correlation analysis of differential gut microorganisms and metabolites

**Fig 5:**
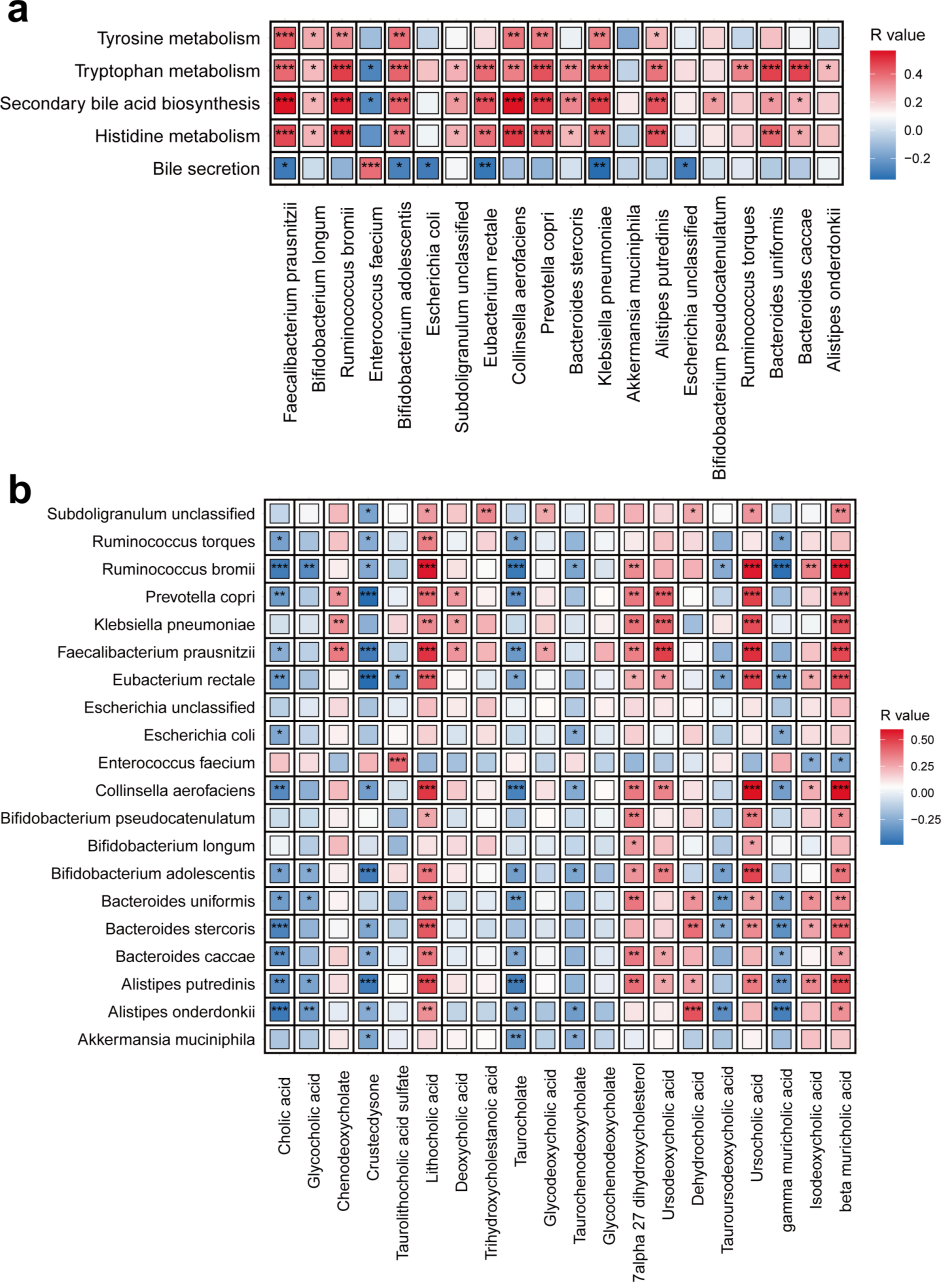
(**a**) Heatmap of the Spearman’s rank correlation of the top 20 most abundant species and altered fecal metabolic pathway. (**b**) Heatmap of the Spearman’s rank correlation of the top 20 most abundant species and 20 bile acids in feces. The statistical significance was denoted inside the squares. *P < 0.05; **P < 0.01; and ***P < 0.001.

Spearman correlation analysis was performed to better understand the relationships between the gut microbiome and fecal metabolites. We found that *Faecalibacterium prausnitzii*, *Bifidobacterium adolescentis*, *Eubacterium rectale*, and *Prevotella copri* were positively correlated with histidine metabolism, secondary bile acid biosynthesis, and tryptophan metabolism, respectively; while the abundance of *Enterococcus faecium* negatively correlated with tryptophan metabolism and secondary bile acid biosynthesis pathways (Fig. 5a). Then, as shown in Fig. 5b, correlation analysis between differential microbiota and bile acids revealed that *Faecalibacterium prausnitzii*, *Bifidobacterium adolescentis*, *Eubacterium rectale*, and *Prevotella copri* positively correlated with ursocholic acid, lithocholic acid, and ursodeoxycholic acid, respectively. These results suggested that reduced abundance of specific gut microbes may be an important cause of homeostatic imbalance in bile acid metabolism in CAP patients.

**Fig 6:**
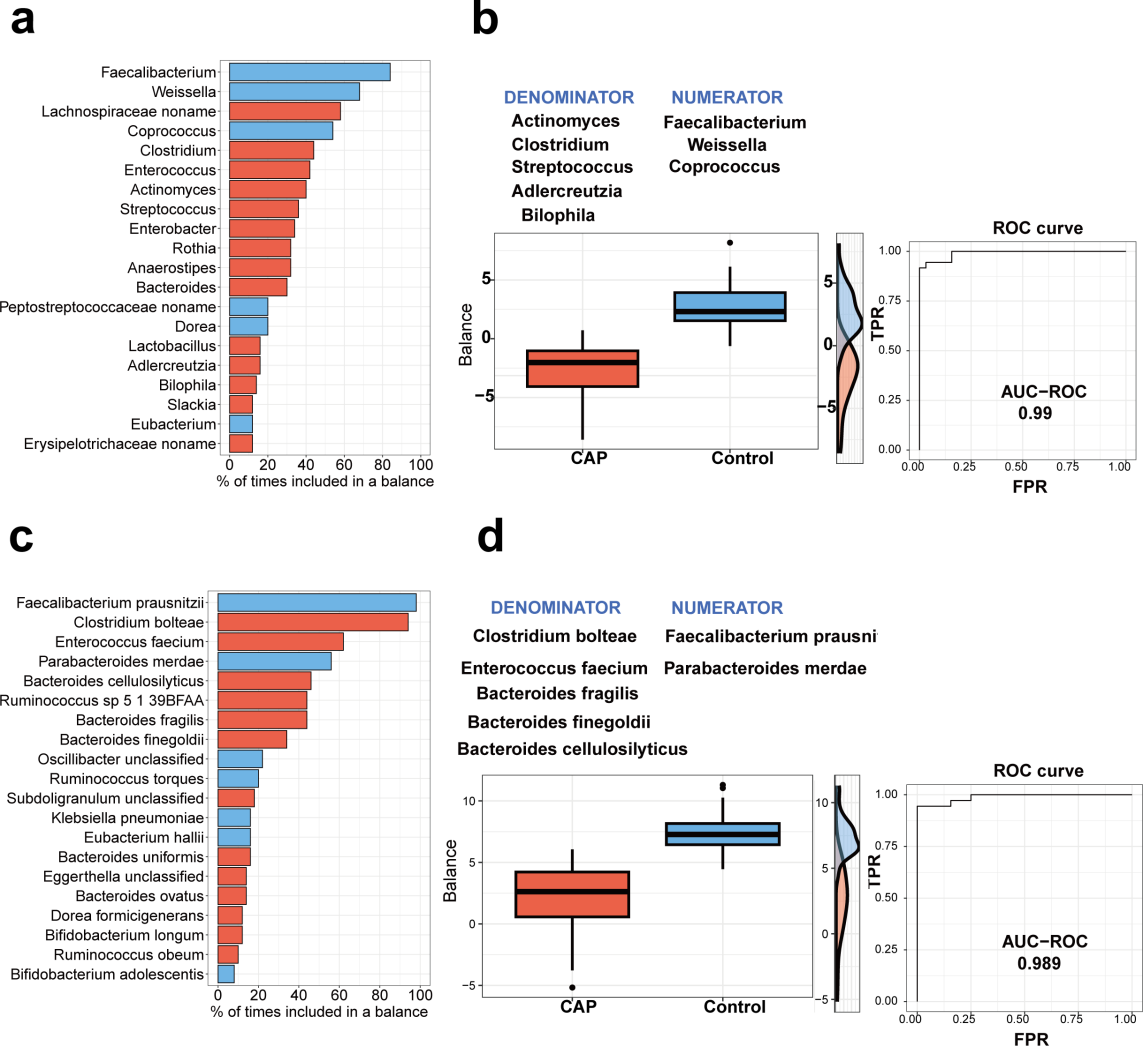
Description of the global balance in gut microbiome for CAP patients. (**a**) The bar chart showing the top 20 intestinal bacterial genera with the highest frequency. (**b**) Balance scores of intestinal bacterial genera for CAP patients (orange color) and control groups (blue color) are shown in the box chart. (**c**) The bar chart showing the top 20 intestinal bacterial species with the highest frequency. (**d**) Balance scores of intestinal bacterial species for CAP patients (orange color) and control groups (blue color) are shown in the box chart.

The goal of selbal analysis is to identify a microbial signature for CAP disease that is able to discriminate between CAP and non-CAP individuals. This microbial signature is defined by two groups of taxa whose relative abundances or balance is associated with CAP disease status. First, we calculated the geometric means of relative abundances of the genera in the gut microbiome between CAP patients and control groups. The results confirm that *Faecalibacterium* was the most frequent species (over 80%) in the balance of the cross-validation between CAP patients and control groups (Fig. 6a). Figure 6b presents the distribution of the microbial signature values for CAP and non-CAP individuals at the genus level. Clearly, patients with CAP disease have lower balance scores than controls, and the balance showed a best-fitting discrimination accuracy (AUC = 0.99). Then, we also calculated the geometric mean of the relative abundances of the species in the gut microbiome between patients with CAP and control groups. The results demonstrate that *Faecalibacterium prausnitzii* was the most frequent species (over 90%) in the balance of the cross-validation between CAP patients and control groups (Fig. 6c). Patients with CAP disease have lower balance scores than controls, and the balance showed a best fitting discrimination accuracy (AUC = 0.989 [Fig. 6d]).

## DISCUSSION

Several previous reports have mainly demonstrated community- or genus-level shifts associated with CAP through 16S rRNA sequencing (24, 25), but the species-level insights and functional multi-omic profiling of gut metagenomes remain poorly characterized. Therefore, we performed an integrated analysis of metagenomic sequencing and fecal metabolomics to explore the role of the gut microenvironment in CAP. In this study, we found that dysbiosis of the gut microbiota can lead to decreased SBAs and insufficient production of SCFAs, which may result in metabolic inflammation in the body.

Our findings demonstrated that the composition of the gut microbiota in CAP patients has already changed compared to the control group. Short-chain fatty acid-producing genera, including *Faecalibacterium*, *Ruminococcus*, and *Eubacterium*, and species, *Faecalibacterium prausnitzii*, *Bifidobacterium adolescentis*, *Eubacterium rectale*, *Prevotella copri*, and *Ruminococcus bromii*, were significantly reduced in CAP patients relative to those in the control group. Our results are consistent with the emerging concept that gut SCFAs remotely affect lung diseases, including asthmatic reactions (26–28) and respiratory infections (29–32). SCFAs, particularly butyrate, have strong immune regulatory properties (33). Liu et al. (34) showed that butyrate can reduce the expression of TLR4 and histone deacetylase and inhibit inflammation in LPS-induced acute lung injury. Butyrate also has the potential to restore IL-10 levels in the lungs by inhibiting histone deacetylases in myeloid-derived suppressor cells, which in turn reduces persistent pulmonary inflammation during infection (35). In addition, the benefits of gut microbiota-associated SCFAs are receiving increasing attention. Haak et al. have demonstrated that the abundance of butyrate-producing microbiota is an independent predictor of lower respiratory viral infection risk in transplant patients (36). A recent study in mice demonstrated that *Bifidobacterium* plays an important role in regulating the immune system to prevent pulmonary inflammation and damage caused by respiratory viruses (37).

Besides, we found the abundance of potential pathobionts was increased in CAP patients, including Enterococcaceae family (*Enterococcus* genus). Lee et al. (38) demonstrated that *Enterococcal* strains in the gut microbiome of CAP patients may become opportunistic and antibiotic-resistant pathogens, shaping a pro-inflammatory environment in the gut. Thus, our results and previous studies suggest that an imbalance in SCFA production may result in metabolic inflammation in the development of CAP.

Disrupted gut microbiota could increase the susceptibility to infections and predispose patients to the development of CAP (36). In recent years, some reports demonstrated that a healthy gut microbiota enhances the antibacterial activity of alveolar macrophages, which is beneficial for lung host defense (14, 39, 40). Disrupted gut microbiota can facilitate the replication of opportunistic pathogenic bacteria in the lungs, such as *Streptococcus pneumoniae* (41). Our study indicates that pro-inflammatory bacteria were over-represented as the core species in CAP patients. *Alistipes putredinis* and *Bacteroides caccae* were more likely to constitute core species of the gut microbiota in CAP patients, which suggests these bacteria were important in the regulation of immunity to CAP.

We found that SBAs, including deoxycholic acid, lithocholic acid, and ursodeoxycholic acid, were significantly reduced. Alterations in bile acid metabolism provided another potential mechanism for the development of CAP. Secondary bile acids, which are the primary ligands for TGR5 (42), have been shown to inhibit pro-inflammatory signaling (7, 43), potentially leading to impaired immunity against viral infections (44). While recent research has linked gut microbiota to the development of CAP (24, 25), the role of bile acid metabolism in this process has often been overlooked. In our study, *Faecalibacterium prausnitzii*, *Bifidobacterium adolescentis*, *Eubacterium rectale*, and *Prevotella copri* positively correlated with histidine metabolism, tryptophan metabolism, and secondary bile acid biosynthesis, respectively, while the abundance of *Alistipes putredinis* negatively correlated with tryptophan metabolism and secondary bile acid biosynthesis pathways. Our data suggest that the interplay between gut microbiota and bile acid metabolism may be a key factor in CAP-related inflammation.

Functional characterization of ARGs in fecal samples was performed using the metagenomic expression libraries, and a total of 210 genes were identified in the gut microbiome (45). ARGs in human gut bacteria can not only be exchanged within the gut microbiota but can also be transferred to other bacteria, thereby increasing the risk of antibiotic resistance in human pathogens (46). Shuai et al. (47) found that a greater diversity of ARGs is associated with a higher risk of type 2 diabetes. However, the effects on CAP were poorly characterized. Thus, in our study, seven differential ARGs, including *AAC(6)-li*, *efmA*, *ErmT*, *msrC*, *tet(L*), *tetM,* and *vanHM,* were significantly higher in CAP patients. Interestingly, Spearman’s correlation coefficient analysis found that *Enterococcus faecium* was positively correlated with the *efmA*, *msrC*, *tet(L*), *tetM,* and *vanHM*, and *ErmT* was positively correlated with *Clostridium bolteae*, suggesting that the transfer of ARGs between microbial communities may be an important pathogenic event for CAP, which is worth further investigation.

This work has several limitations. First, our study only recruited local CAP patients in Zibo, and patients enrolled in this study were mainly elderly with a median age of 65.8. Therefore, it is necessary to validate more extensive and diverse populations in the future. Second, this study lacks severe diseases, which limits our ability to classify or predict the progression of CAP. So, further improvements, such as increasing the sample size and designing studies on different severity stages of CAP, are needed. Third, no bacterial strain was isolated to validate the mechanism in animal models. Further research must explore the underlying mechanism between the altered gut microbiota and metabolites of CAP in animal models.

In conclusion, this study characterized the gut microecology and metabolite profiles of patients with CAP compared to a control group. We identified bile acid insufficiency, imbalanced SCFA production, and excessive metabolic inflammation as gut microbiota-driven factors that contribute to CAP development. These findings provide insights into the mechanisms linking altered gut microbiota to CAP pathogenesis and suggest that targeting the gut microbiota may be a promising strategy for intervening in CAP.

## Data Availability

The data sets presented in this study can be found in online repositories. The names of the repository/repositories and accession number(s) are as follows: https://www.ncbi.nlm.nih.gov/, PRJNA1152927 (metagenomic) and https://ngdc.cncb.ac.cn/, PRJCA034666 (metabolomic).

## SUPPLEMENTAL MATERIAL

Data S1 spectrum.02639-24-s0001.xlsx List of 198 pathogens detected by tNGS.

Data S2 spectrum.02639-24-s0002.xlsx List of 452 significantly altered metabolites.

Data S3 spectrum.02639-24-s0003.xlsx List of 106 differential expression metabolites.

Fig. S1 spectrum.02639-24-s0004.docx The gut microbial species in the sub-network structure.

Supplemental material spectrum.02639-24-s0005.docx Supplemental data legends.

Table S1 spectrum.02639-24-s0006.docx Clinical characteristics of enrolled patients and controls.

ASM does not own the copyrights to Supplemental Material that may be linked to, or accessed through, an article. The authors have granted ASM a non-exclusive, world-wide license to publish the Supplemental Material files. Please contact the corresponding author directly for reuse.
