# Supplementary material for: Altered gut microbiota and metabolite profiles in community-acquired pneumonia: a metagenomic and metabolomic study
Source: Microbiol Spectr. 2025 Mar 10;13(4):e02639-24. doi: 10.1128/spectrum.02639-24 (PMC11960049; doi:10.1128/spectrum.02639-24)
Supplement: Fig. S1 — The gut microbial species in the sub-network structure. [file spectrum.02639-24-s0004.docx]

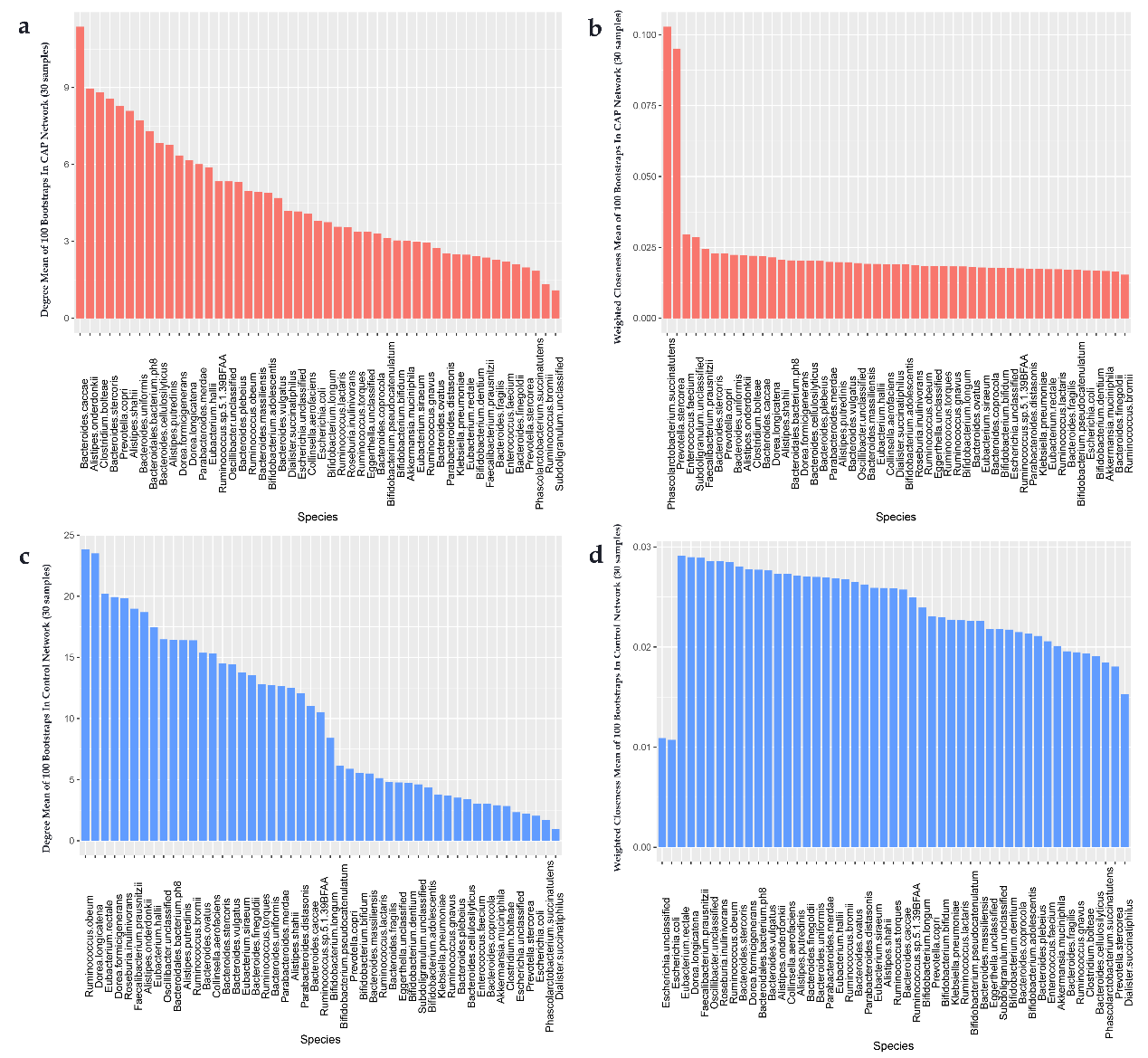


**Supplementary Figure 1.** The gut microbial species in the sub-network structure. Means of the weighted degree of 100 sub-networks from CAP (a) and Control (c). Means of the weighted closeness of 100 sub-networks from CAP (b) and Control (d). The sub-network was constructed in randomly selected 30 samples from both CAP and Control group. This procedure was repeated 100 times then we got 100 sub-networks from each group to calculate the average of weighted degree and weighted closeness of gut microbial species.
