# Supplementary material for: Altered gut microbiota and metabolite profiles in community-acquired pneumonia: a metagenomic and metabolomic study
Source: Microbiol Spectr. 2025 Mar 10;13(4):e02639-24. doi: 10.1128/spectrum.02639-24 (PMC11960049; doi:10.1128/spectrum.02639-24)
Supplement: Supplemental material — Supplemental data legends. [file spectrum.02639-24-s0005.docx]

**Supplemental data 1.** List of 198 pathogens detected by targeted next-generation sequencing (tNGS).

**Supplemental data 2.** List of 452 significantly altered metabolites.

**Supplemental data 3.** List of 452 106 differential expression metabolites.
