# Supplementary material for: Altered gut microbiota and metabolite profiles in community-acquired pneumonia: a metagenomic and metabolomic study
Source: Microbiol Spectr. 2025 Mar 10;13(4):e02639-24. doi: 10.1128/spectrum.02639-24 (PMC11960049; doi:10.1128/spectrum.02639-24)
Supplement: Table S1 — Clinical characteristics of enrolled patients and controls. [file spectrum.02639-24-s0006.docx]

**Supplementary Table 1.** Detailed pathogen information of participants.

|  | **Group** | **Name** | **Sample** | **Pathogen** | | |
| --- | --- | --- | --- | --- | --- | --- |
|  |  |  |  | **Virus** | **Bacteria** | **Fungus** |
| 1 | CAP patients | PF01LLC1 | oropharyngeal swab | SARS-CoV-2 |  |  |
| 2 | CAP patients | PF02CGL3 | oropharyngeal swab | SARS-CoV-2 | Acinetobacter baumannii |  |
| 3 | CAP patients | PF02KHQ1 | oropharyngeal swab |  |  |  |
| 4 | CAP patients | PF05GXY1 | Sputum | SARS-CoV-2 | Pseudomonas aeruginosa |  |
| 5 | CAP patients | PF06WXZ1 | oropharyngeal swab | SARS-CoV-2 | Acinetobacter baumannii |  |
| 6 | CAP patients | PF07LHW1 | Sputum | SARS-CoV-2 | Streptococcus pneumoniae | Aspergillus fumigatus |
| 7 | CAP patients | PF08CXM1 | Sputum | SARS-CoV-2 |  |  |
| 8 | CAP patients | PF08CXYF | Sputum | SARS-CoV-2 | Acinetobacter baumannii  Pseudomonas aeruginosa |  |
| 9 | CAP patients | PF08FWZ3 | Sputum | SARS-CoV-2 | Klebsiella pneumoniae |  |
| 10 | CAP patients | PF13CCL1 | Sputum | SARS-CoV-2 |  |  |
| 11 | CAP patients | PF15ZWM1 | Sputum | SARS-CoV-2 |  |  |
| 12 | CAP patients | PF16LFM1 | Sputum | SARS-CoV-2 |  | Aspergillus fumigatus |
| 13 | CAP patients | PF16LPX3 | oropharyngeal swab |  |  |  |
| 14 | CAP patients | PF19YFC3 | Sputum | SARS-CoV-2 | Haemophilus influenzae |  |
| 15 | CAP patients | PF19ZYJ1 | Sputum | SARS-CoV-2 | Klebsiella pneumoniae  Acinetobacter baumannii  Streptococcus pneumoniae |  |
| 16 | CAP patients | PF21WHA1 | Sputum |  | Staphylococcus aureus |  |
| 17 | CAP patients | PF26ZCM1 | Sputum |  | Staphylococcus aureus |  |
| 18 | CAP patients | PF28CZP1 | Sputum | SARS-CoV-2 |  |  |
| 19 | CAP patients | PF29XHJ1 | oropharyngeal swab |  |  |  |
| 20 | CAP patients | PF30LTZ1 | Sputum | SARS-CoV-2 |  |  |
| 21 | CAP patients | PF33SGY1 | Sputum | SARS-CoV-2 | Klebsiella pneumoniae  Acinetobacter baumannii |  |
| 22 | CAP patients | PF34HKM1 | Sputum |  |  |  |
| 23 | CAP patients | PF35WYZ1 | Sputum |  |  |  |
| 24 | CAP patients | PF36YRS1 | Sputum |  |  |  |
| 25 | CAP patients | PF41DYP1 | Sputum | SARS-CoV-2 |  |  |
| 26 | CAP patients | PF41ZBX3 | Sputum |  | Klebsiella pneumoniae  Haemophilus influenzae |  |
| 27 | CAP patients | PF45CXZ1 | oropharyngeal swab |  |  |  |
| 28 | CAP patients | PF46SAQ1 | Sputum |  |  | Rhizopus oryzae |
| 29 | CAP patients | PF47YJM1 | Sputum |  | Haemophilus influenzae |  |
| 30 | CAP patients | PF48WGY3 | oropharyngeal swab | SARS-CoV-2 |  |  |
| 31 | CAP patients | PF48XSQ1 | Sputum | SARS-CoV-2 |  |  |
| 32 | CAP patients | PF52LMD1 | Sputum |  |  |  |
